# Patient motion tracking for non‐isocentric and non‐coplanar treatments via fixed frame‐of‐reference 3D camera

**DOI:** 10.1002/acm2.12842

**Published:** 2020-02-28

**Authors:** Sergey Gasparyan, Kyle Ko, Lawrie B. Skinner, Ryan B. Ko, Billy W. Loo, Benjamin P. Fahimian, Amy S. Yu

**Affiliations:** ^1^ Department of Radiation Oncology Stanford university Palo Alto CA 94304 USA

**Keywords:** intrafractional monitoring, non‐coplanar, non‐isocentric, optical surface monitoring

## Abstract

**Purpose:**

As C‐arm linac radiation therapy evolves toward faster, more efficient delivery, and more conformal dosimetry, treatments with increasingly complex couch motions are emerging. Monitoring the patient motion independently of the couch motion during non‐coplanar, non‐isocentric, or dynamic couch treatments is a key bottleneck to their clinical implementation. The goal of this study is to develop a prototype real‐time monitoring system for unconventional beam trajectories to ensure a safe and accurate treatment delivery.

**Methods:**

An in‐house algorithm was developed for tracking using a couch‐mounted three‐dimensional (3D) depth camera. The accuracy of patient motion detection on the couch was tested on a 3D printed phantom created from the body surface contour exported from the treatment planning system. The technique was evaluated against a commercial optical surface monitoring system with known phantom displacements of 3, 5, and 7 mm in lateral, longitudinal, and vertical directions by placing a head phantom on a dynamic platform on the treatment couch. The stability of the monitoring system was evaluated during dynamic couch trajectories, at speeds between 10.6 and 65 cm/min.

**Results:**

The proposed monitoring system agreed with the ceiling mounted optical surface monitoring system in longitudinal, lateral, and vertical directions within 0.5 mm. The uncertainty caused by couch vibration increased with couch speed but remained sub‐millimeter for speeds up to 32 cm/min. For couch speeds of 10.6, 32.2, and 65 cm/min, the uncertainty ranges were 0.27– 0.73 mm, 0.15–0.87 mm, and 0.28–1.29 mm, respectively.

**Conclusion:**

By mounting a 3D camera in the same frame‐of‐reference as the patient and eliminating dead spots, this proof of concept demonstrates real‐time patient monitoring during couch motion. For treatments with non‐coplanar beams, multiple isocenters, or dynamic couch motion, this provides additional safety without additional radiation dose and avoids some of the complexity and limitations of room mounted systems.

## 1 INTRODUCTION

With the development of digital LINACs, advanced radiation therapy techniques with non‐coplanar and non‐isocentric beams, as well as beams with dynamic couch motion are emerging. These methods such as station parameter optimized radiation therapy (SPORT), 4‐Pi, trajectory optimization in radiotherapy using sectioning (TORUS), trajectory modulated arc therapy (TMAT), and HyperArc^TM^ 1, 2, 3, 4, 5 offer enhanced dosimetry and more efficient treatments. Patient position monitoring during these new techniques is a key bottleneck to clinical implementation. Specifically, non‐coplanar and non‐isocentric beam arrangements prohibit the use of gantry mounted on‐board imaging systems. Although the ceiling mounted x‐ray systems can track patient position, they are limited to isocentric treatments.^6^, ^7^ Optical surface monitor systems, such as OSMS, AlignRT, C‐RAD, and humediQ, are some of the solutions for non‐coplanar treatments.^8^ However, there are a few known issues with those surface imaging systems: (a) the systems are calibrated to accurately monitor the patient around the isocenter and may not be accurate for non‐isocentric treatments, (b) some systems require manual couch angle input, which complicates dynamic couch treatments, (c) increased uncertainty occurs when one or more cameras are blocked by the gantry (blind spots),^9^ and (d) the inaccuracy of the optical system increases for couch rotations, due to misalignment effects during the calibration process.^10^ This proposed couch‐mounted system allows the tracking data to be unaffected by the movement of the couch, blind spots and allows easy calibration due to the fact that a three‐dimensional (3D) camera is in the same frame‐of‐reference as the patient.

To truly benefit from complex arc deliveries or dynamic couch movement,^11^ an efficient and reliable monitoring system must be in place. With computer vision, it is feasible to simultaneously monitor patient position and ensure the treatment beam delivery. The goal of this study is to develop a real‐time patient position monitoring system for advanced unconventional beam trajectories to ensure a safe and accurate treatment delivery with the camera on the couch. By putting the depth sensor in the same frame‐of‐reference as the patient, that is, camera on the couch, all the issues mentioned above become resolvable.

## 2 MATERIALS AND METHODS

There are a range of commercially available depth cameras suitable for this purpose. In this work, a Kinect v2 depth camera (Microsoft, Redmond, WA, USA) was used for the patient position monitoring system. The camera uses infrared laser projectors and a monochrome CMOS sensor to measure the depth via the time of flight technique, that is, the time between sending and receiving IR pulses is converted to depth for each location on the 2D CMOS sensor.^12^ The Kinect v2 camera has a maximum frame rate of 60 Hz and a depth range from 0.5 to 4.5 m. The firmware allows a desired depth range to be resolved into a maximum of 768 depth values. This gives voxel depth resolution from 0.68 to 5.8 mm. For our setup with 0.8 m depth range, the voxel depths are approximately 1 mm. The proposed monitoring system is being used with a relative concept, so the absolute isocenter position calibration is not required — the absolute distance between the patient and the camera does not change during dynamic couch treatments, so the tracking accuracy will not degrade while the couch is moving. A check of the scaling and orientation of axes is needed for these relative measurements. The tracking system starts by storing the first 60 frames. After this phase of the algorithm is complete, displacement readings will begin to output to the user. With this proposed system, the calibration is simplified because the 3D camera is in the same frame‐of‐reference as the patient which makes it independent of couch movement.

### 2.1 An algorithm to acquire the real‐time point cloud

An algorithm was developed to acquire the surface point cloud as the reference image and compare to real‐time surface images with the 3D camera. The region class was implemented to store all the depth information in the region that is tracked. Specifically, it contains an array holding the x, y, and z values of each voxel in the specified region. These x, y, and z values are encapsulated in a Point class. The software uses this region class to take a snapshot of the x, y, and z values of every voxel in the region. The software then uses this data to track the lateral, longitudinal, and vertical displacements of the specified object. A standard coordinate transformation matrix was applied to the x, y, and z from the camera into the x', y', and z' of the couch. The x and y coordinates represent the location on the grid of pixels that each pixel is located at. The z represents each pixel distance from the depth sensor. By specifying the region of interest to be tracked by the software, for example, patient face, a circular region will form around the clicked point and the depth readings of the voxels in that region‐of‐interest will be stored in a Region object. The monitoring region of interest can be set by the user of the program from a few mm to the full field of view of the camera (58 × 46°), which allows imaging of objects over 1 m in length and width at the 1.5‐m camera distance. To reduce noise, the x, y, and z positions of each voxel in this region are then averaged over 60 frames. After the reference region is created, a region called Comp will be created on every iteration of the program. This region will be compared to the reference region and displacement values were calculated. Comp is the live feed of pixels that comparing with the reference pixels to figure out the displacement of the object.

### 2.2 Accuracy of patient position monitoring system

The software finds a unique voxel in the reference region by finding the voxel with the closest depth value to the camera. Voxels with depth values within 3 mm of the unique voxel were then identified and tracked. The depth can be adjusted to be larger or smaller if the region being tracked is something as intricate as a face or as smooth as a shoulder. This could be decided before treatment. The x, y, and z values of all the tracked voxels, in the reference region were then averaged. The same process was repeated to compute the Comp region. The Comp and reference regions were then compared by computing the average x, y, and z values of each region. To reduce variance in the reading, for each reading the algorithm takes 60 frames and averages the difference between the reference image and the live image. This process occurred 60 times over 1 s. For every trial, 10 readings of the Kinect were acquired and averaged to get the displacement values.

**Figure 1:**
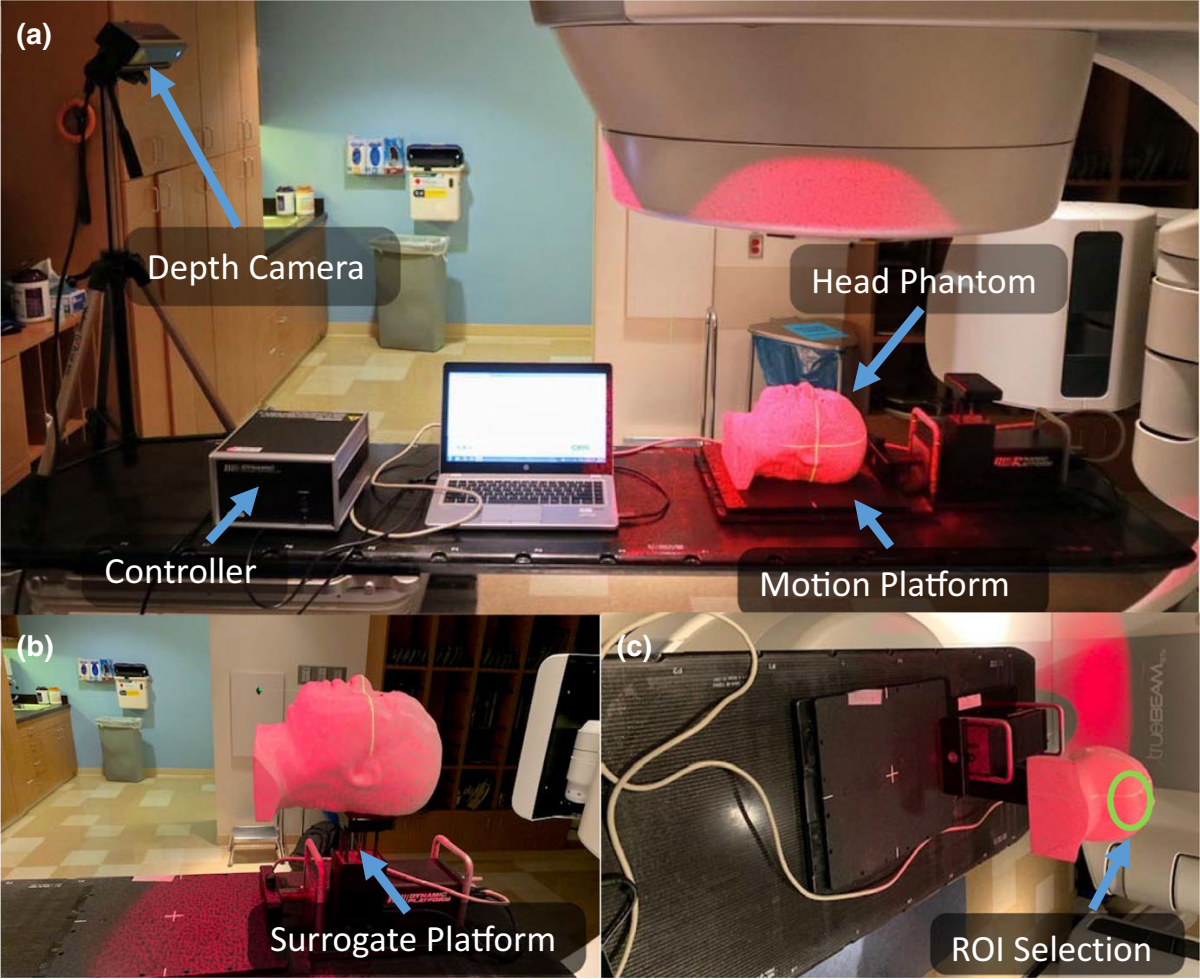
The monitoring accuracy of the developed software is compared to the commercial optical surface monitoring system. (a) The overview of the experimental setup. The head phantom is on the motion platform for lateral and longitudinal shifts. (b) The head phantom is on the surrogate platform for vertical shifts. (c) The region of interest (ROI) selection from the view of depth camera.

The accuracy of patient position monitoring system was evaluated by moving the 3D printed patient phantom on the couch. The phantom was shifted by known displacements using a dynamic platform (Computerized Imaging Reference Systems, Inc., Norfolk, VA, USA) of the 3, 5, and 7 mm in lateral, longitudinal, and vertical directions. The Kinect was set up at the end of the treatment table and pointed at the phantom. The accuracy of the software was evaluated against the optical surface monitoring system (OSMS, Varian, Palo Alto, CA) which is an optical surface mapping system that uses a 3D point cloud to represent the surface of subject's body obtained from a 3D camera in‐room monitoring system and compares it with a CT‐derived surface as a reference, imported via a DICOM file from the treatment planning system. The measurements were repeated five times. The agreement between the known shift from the OSMS and displayed surface deltas from the proposed system were evaluated to define a reliable monitoring for treatments. The experimental setup is shown in Fig. 1.

### 2.3 Evaluation of uncertainty of surface monitoring system with dynamic movement

Dynamic couch treatment plans (.xml file) for a non‐coplanar treatment were created and delivered in Varian Developer Mode (Varian Medical Systems, Palo Alto, CA, USA). The phantom was monitored by the camera on the couch monitoring system. The developed software was used to monitor phantom surface and compared it to the predefined reference image during the dynamic couch treatment in real time. The updated deltas, which are ideally zero since no extra phantom shift was introduced, were used to determine the uncertainty of the system during couch vibration and acceleration. For every trial, readings of the Kinect were taken over the region of the interest and averaged to get the displacement values. The couch was moved in lateral, longitudinal, and vertical directions, as well as rotated with different speeds. The speed of the couch was controlled by keeping the MU and couch displacement fixed and varying the dose rate (100 MU/min, 300 MU/min and 600 MU/min) of the plan.

## 3 RESULTS

### 3.1 Monitoring accuracy

Table 1 shows the 3D camera measured lateral, longitudinal, and vertical phantom displacements for planned shifts from 3 to 7 mm. All the Kinect measured shifts were within 0.4 mm of the OSMS system. The overall averaged delta between OSMS and Kinect displacement measurements was 0.2 mm in each direction. The software values have larger standard deviations and then the OSMS values.

**Table 1:** The monitoring accuracy of the proposed monitoring system.

n = 5	Lateral shift			Longitudinal shift			Vertical shift		
	3 mm	5 mm	7 mm	3 mm	5 mm	7 mm	3 mm	5 mm	7 mm
Mean ± SD, mm (Kinect)	2.42 ± 0.10	4.95 ± 0.11	6.52 ± 0.30	2.70 ± 0.13	4.64 ± 0.08	7.00 ± 0.19	3.14 ± 0.31	5.10 ± 0.14	6.94 ± 0.19
Mean ± SD, mm (OSMS)	2.82 ± 0.04	4.86 ± 0.05	6.80 ± 0.00	2.86 ± 0.05	4.74 ± 0.05	6.80 ± 0.07	2.94 ± 0.05	4.84 ± 0.05	6.86 ± 0.13
Delta, mm	0.40	‐0.09	0.28	0.16	0.10	‐0.20	‐0.20	‐0.26	‐0.08

### 3.2 Uncertainty with dynamic couch movement

The tracking delta was measured at different couch speeds while the phantom is stationary on the couch (Table 2), that is, the relative position of the 3D camera and the phantom is constant while the couch is moving. As expected, the accuracy decreases with increasing couch speed. The uncertainty ranged from 0.27 to 0.73 mm, 0.15 to 0.87 mm, and 0.28 to 1.29 mm for couch speeds 10.6, 32.2, and 65 cm/min, respectively. Overall, sub‐mm accuracy is maintained, except the highest speed, 65 cm/min, where lateral displacement was 1.29 mm.

**Table 2:** The uncertainty of the proposed monitoring system.

Speed (cm/min)	Delta (mm)			
	Longitudinal	Lateral	Vertical	Rotational
10.6	0.27	0.73	0.18	0.43
32.2	0.36	0.87	0.15	0.72
65.0	0.28	1.29	0.55	0.71

## 4 DISCUSSION

The overarching goal of this study is to develop a real‐time monitoring technique for treatments with unconventional beam trajectories to ensure safe and accurate delivery. A couch‐mounted depth camera offers fixed frame‐of‐reference tracking of patient motion independent of couch and gantry positions. This is projected to become yet more significant as gantry‐based linac techniques strive to improve dosimetry by including complex couch motion.

For the accuracy of the software, the lateral displacement was more difficult to track due to the fact that the depth sensor does not intrinsically calculate lateral displacement. The software approximates lateral displacement using the movement of the pixels being tracked, unlike longitudinal displacement which it calculates using the fluctuations of the depth values of the pixels. The longitudinal displacement can be more readily calculated because the depth sensor is created for tracking longitudinal displacements. The decrease in monitoring accuracy with increasing couch speed is likely caused by a combination of couch vibration and flexure of the camera mount under acceleration.

Non‐coplanar and non‐isocentric treatments provide promising dosimetric results, however, without intra‐fractional monitoring, it is hard to ensure the patient position throughout treatment. During treatments, the couch will be rotated for non‐coplanar beams or moved away from the isocenter to treat extended volumes. Even for currently available surface imaging techniques such as OSMS and C‐RAD, there is reduced accuracy for couch rotations. These inaccuracies are caused by the misalignment of the calibration plate during the calibration process.^10^ By mounting a 3D camera in the same frame‐of‐reference as the patient provides a novel and feasible way to monitor patient position during the treatment. It not only eliminates the complicated calibration process but also the blind spots caused by gantry and the on‐board imager systems. The continuous patient position monitoring afforded by a couch mounted camera can provide confidence that the planned dose is accurately delivered during the whole treatment.

**Figure 2:**
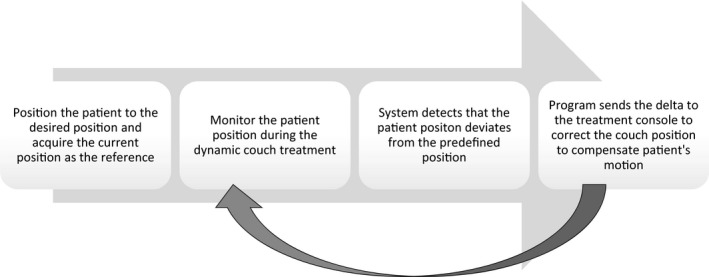
The future flowchart of adjusting and correcting the couch position to the desired position when the patient position is out of the tolerance compared to its reference.

For future work, it is not only critical to know if the patient position deviates from the plan, but also to send the correcting shift back to the treatment console. Through collaboration with vendors, it is feasible that the program will send out the delta to correct the patient position to the treatment console in real time *via* the motion management interface (Fig. 2). Collision of the machine with the patient during treatment, however, is still unsolved for these non‐isocentric, and non‐coplanar treatments. Most studies focus on collision prediction of the treatment plan, but not real‐time monitoring.^13^, ^14^ Since the camera is on the couch, with the combination of the 3D computer‐aided design of the linac, it is feasible to build a real‐time collision avoidance system — if we know the location of patient relative to the machine, the collision can be avoided during the treatment.

## 5 CONCLUSION

An affordable system for monitoring advanced non‐coplanar, non‐isocentric, and dynamic couch treatment strategies is demonstrated. A motion tracking software with a camera mounted to the treatment table was designed and evaluated. By putting a depth sensor in the patients’ frame‐of‐reference, dead spots can be eliminated. With this system, real‐time surface monitoring during complex treatments with dynamic couch motion is feasible.

## CONFLICT OF INTERESTS

Authors declare no financial or other relationships, which may lead to a conflict of interest.
